# *Neoethilla*, a new genus for the first record of the Ethillini from the New World (Diptera, Tachinidae, Exoristinae)

**DOI:** 10.3897/zookeys.242.3974

**Published:** 2012-11-15

**Authors:** Pierfilippo Cerretti, D. Monty Wood, James E. O’Hara

**Affiliations:** 1 Dipartimento di Biologia e Biotecnologie “Charles Darwin”, Università di Roma “La Sapienza”, Piazzale A. Moro 5, 00185, Rome, Italy; 2 Centro Nazionale per lo Studio e la Conservazione della Biodiversità Forestale – Corpo Forestale dello Stato, Via Carlo Ederle 16/A, 37100, Verona, Italy; 3 Canadian National Collection of Insects, Agriculture and Agri-Food Canada, 960 Carling Avenue, Ottawa, Ontario, Canada K1A 0C6

**Keywords:** Ethillini, *Exorista ignobilis* van der Wulp, Nearctic Region, Neotropical Region, *Winthemia antennalis* Coquillett, new genus, new synonymy, phylogeny, systematics, Winthemiini

## Abstract

New genus *Neoethilla* **gen. n.**, is described to include two New World nominal species formerly recognized as valid species in *Winthemia* Robineau-Desvoidy: *Exorista ignobilis* van der Wulp and *Winthemia antennalis* Coquillett. *Winthemia antennalis* is proposed as a junior synonym of *Exorista ignobilis* **syn. n.** *Neoethilla ignobilis* **comb. n.** is removed from the Winthemiini and placed in the tribe Ethillini (Exoristinae) based on a study of the external features of adults, male terminalia, female reproductive system, and egg morphology. The small tribe Ethillini, not hitherto known from the New World, currently comprises fourteen genera worldwide. The phylogeny and systematics of the Ethillini and their relationships with related tribes are discussed and documented by descriptions and illustrations of relevant character states.

## Introduction

Van der Wulp (1890) described *Exorista ignobilis* from Guerrero, Mexico, based on a single male. The species was subsequently moved to *Winthemia* Robineau-Desvoidy, 1830, by Reinhard (1931) and has continued to be treated as a valid species of *Winthemia* to the present day (Guimarães 1972). However, Reinhard (1931) misidentified the species; he did not examine the holotype of *Exorista ignobilis* but instead relied on notes taken of it by J.M. Aldrich in 1929. Based on these notes, Reinhard (1931) misidentified three specimens from Chile and Argentina as *Winthemia ignobilis* and redescribed the species from this material. Aldrich (1934) himself accepted the identifications of Reinhard (1931) and included the species under the same combination in his faunal treatment of the Tachinidae of Patagonia and South Chile. Cortés and Hichins (1969) also recognized a Chilean species of *Winthemia* as *Winthemia ignobilis*. Although the identities of these specimens from Chile and Argentina have yet to be clarified, we suspect that everything once called *Winthemia ignobilis* from these countries is *Winthemia reliqua* Cortés & Campos, 1971.

A second nominal species, *Winthemia antennalis*, was later described by Coquillett (1902) based on a single female from Los Angeles County, California, United States. Coquillett (1897) had earlier misidentified this single specimen as *Winthemia nigrifacies* (Bigot, 1889) in his key to the species of *Winthemia* of America north of Mexico. Tothill (1912) continued the placement of this species in *Winthemia* in his key to the North American species of the genus, as did Reinhard (1931) in his revision of the “American” species of *Winthemia*. This classification was also followed by Sabrosky and Arnaud (1965) in the *Catalog of the Diptera of America north of Mexico*. Guimarães (1972), however, removed *Winthemia antennalis* from *Winthemia* in his revision of the *Winthemia* of America north of Mexico. Without further explanation, he wrote in his abstract: “*Winthemia antennalis* Coquillett does not belong in this genus and its correct placement has not been determined” (Guimarães 1972: 27). Sabrosky (1973) apparently agreed, as he did not include *Winthemia antennalis* in his paper on the identification of *Winthemia* species of America north of Mexico.

*Winthemia antennalis* was again treated as a *Winthemia* species, albeit provisionally, by Wood (1987). Wood (1987: 1210) explained this placement in a footnote: “Although arrangement of postpronotal bristles is different from other species of *Winthemia*, the male terminalia and unembryonated planoconvex egg suggest a relationship, which may ultimately be resolved by additional study of the tribe Winthemiini”. Recently, O’Hara and Wood (2004) maintained this classification and listed *Winthemia antennalis* under *Winthemia* in their catalogue of the Tachinidae of America north of Mexico.

The enigmatic placement and identity of *Winthemia ignobilis* and *Winthemia antennalis* became a topic of discussion among us when several specimens provisionally identified as *Winthemia antennalis* were collected in the Gila National Forest of New Mexico, United States, during the field meeting of the North American Dipterists Society in 2007. Upon further study we have determined that *Winthemia antennalis* Coquillett is a junior synonym of *Exorista ignobilis* van der Wulp and that this taxon belongs not to the Winthemiini but to the Ethillini, a tribe hitherto unknown from the New World. We discuss below the characteristics of the Ethillini and propose a new genus for the single known New World species of this tribe.

## Materials and methods

### Specimens

**Figure 1.**
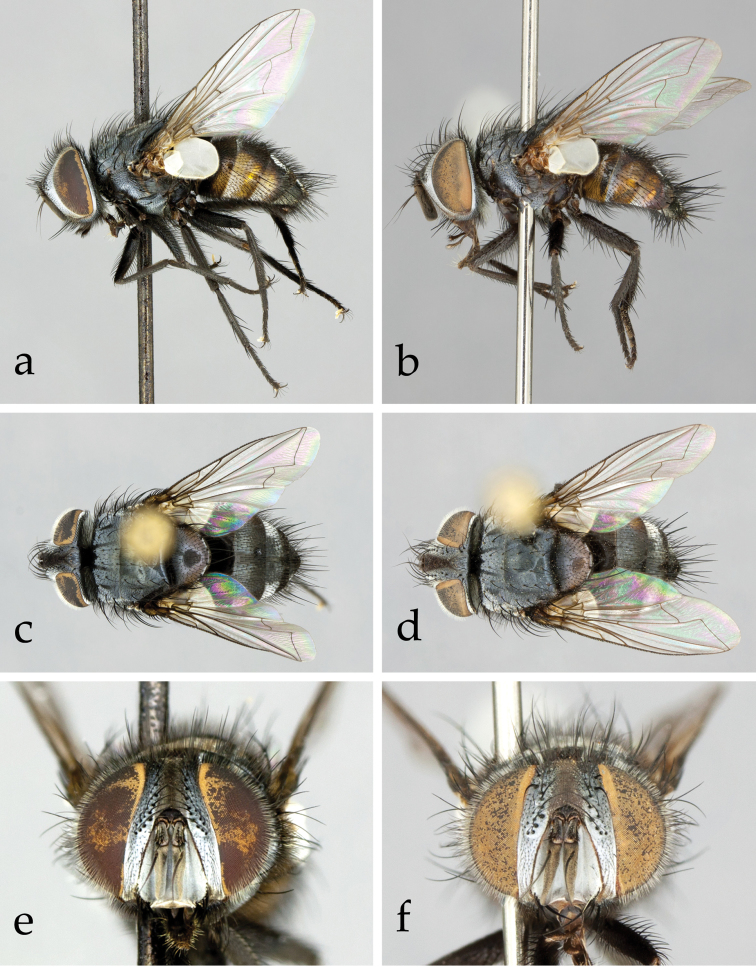
*Neoethilla* gen. n.*ignobilis* (New Mexico) **a–d** habitus **a** male in lateral view **b** female in lateral view **c** male in dorsal view **d** female in dorsal view **d–e** head in frontal view **d** male **e** female.

Male terminalia of *Neoethilla ignobilis* were dissected following the method described in detail by O’Hara (2002), then dehydrated with ethanol and critical point dried. After examination, the terminalia were rehydrated and preserved in glycerine in a plastic microvial pinned below the specimen (cf. Cerretti and Shima 2011). One egg, almost entirely exposed but still attached to the ovipositor of a dried female of *Neoethilla ignobilis* from Plymouth, Massachusetts (CNC), was removed by tilting it carefully with a pin and then mounted on a micropin. After ESEM examination the egg was glued on a tag and pinned below the source specimen. Male terminalia, pinned specimens and egg were examined, uncoated, with a Hitachi TM1000 environmental scanning electron microscope (ESEM); male terminalia were also slide mounted and examined with a Leica DMLS. Figures 1a–e were prepared from composites of images captured using a Canon EOS 40D Digital SLR camera body, with a Canon MP-E 65mm 1–5X macro lens, mounted on a Kaiser RS1 copy stand (for further details see O’Hara 2012); figures 5a–c were prepared from composites of images captured with a Nikon Digital Sight DS-L1, DS-5M mounted on a Leica DMLS. Specimens examined are preserved in the following collections (acronyms used in the text):

BMHN Natural History Museum [formerly British Museum (Natural History)], London, United Kingdom (N. Wyatt).

CNC Canadian National Collection of Insects, Agriculture and Agri-Food Canada, Ottawa, Canada.

MZUR Museum of Zoology, Università degli Studi di Roma “La Sapienza”, Rome, Italy (A. Vigna Taglianti).

USNM National Museum of Natural History [formerly United States National Museum], Smithsonian Institution, Washington, United States (N.E. Woodley).

Label data of the holotypes of *Exorista ignobilis* and *Winthemia antennalis* are cited verbatim with the end of lines and labels indicated by the following symbols: /, end of a line and beginning of the next; //, end of a label and beginning of the next (from top to bottom on the same pin).

## Terminology

Morphological terminology generally follows McAlpine (1981), except for the antenna and a few details of thoracic chaetotaxy for which we are following Stuckenberg (1999) and Tschorsnig and Richter (1998), respectively. Measurements and ratios of the head follow Cerretti (2010).

## Systematics

### Neoethilla gen. n.

urn:lsid:zoobank.org:act:2863EDC7-20B9-4102-AD19-4EF4CFFA6120

http://species-id.net/wiki/Neoethilla

**Figure 2.**
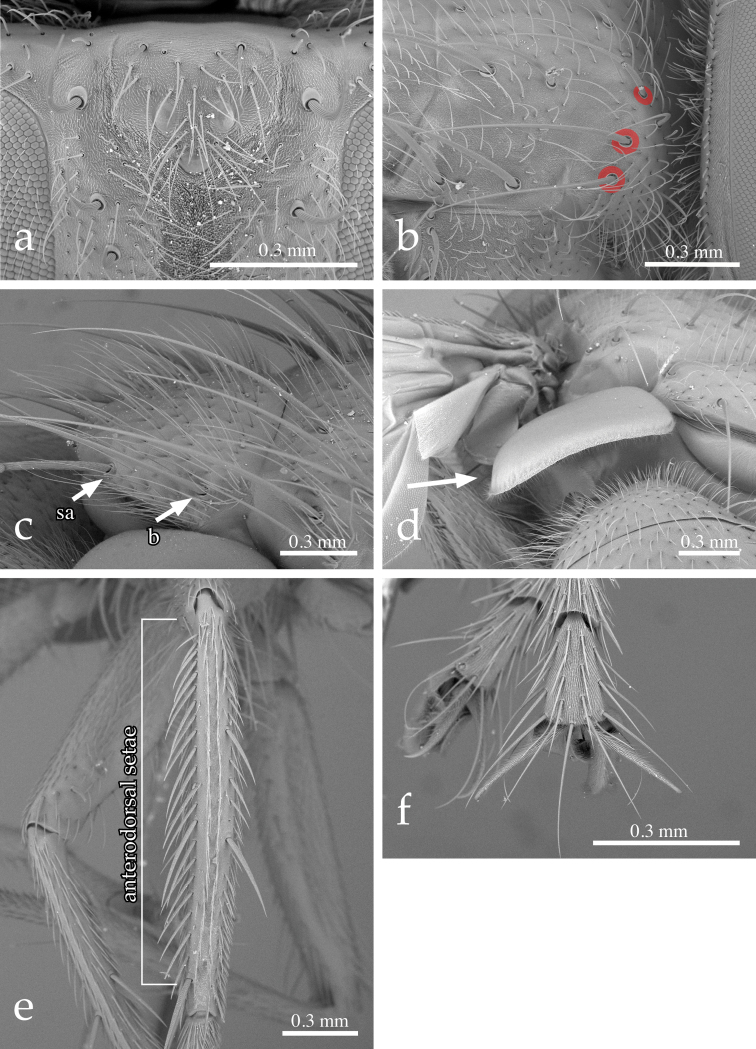
*Neoethilla* gen. n.*ignobilis* (male, New Mexico) **a** vertex in anterodorsal view **b** right postpronotum and part of presutural portion of scutum in laterodorsal view [circles indicate basal postpronotal saetae] **c** scutellum in laterodorsal view [b = basal scutellar seta; sa = subapical scutellar seta] **d** lower calypter in posterior view **e** left hind tibia in dorsal view **f** fore claws.

Figs 1a–f , 2a–b , 4a–d , 5a–e

#### Type species:

*Exorista ignobilis* van der Wulp, 1890, by present designation.

#### Etymology.

The compound name *Neoethilla* refers to the New (Latin, *neo*) World distribution and to the suspected close relationship of this genus with the Old World genus *Ethilla* Robineau-Desvoidy, 1863.

#### Description.

This generic description is based on a redescription of the single included species, *Neoethilla ignobilis*.

Length: 5.5–7.5 mm.

Colour: Head mainly black, covered with grey microtomentum. Palpus black to brown (usually paler in female). Thorax and legs entirely black. Abdomen mainly black but reddish yellow laterally (Fig. 1a–b). Tegula and basicosta black.

Head (Figs 1a–e, 2a): Large in dorsal view, about as wide as thorax; higher than long in lateral view. Compound eye densely covered with long ommatrichia (Fig. 1c–f). Frons at its narrowest point 2/3–6/7 (♂♀) as wide as eye in dorsal view (no significant sexual dimorphism in examined specimens). Frontal vitta (= interfrontal area) clearly widening anteriorly. Outer (= lateral) vertical seta not differentiated from postocular setae in male, well developed in female. Ocellar seta absent (Figs 1c–d, 2a) or very small; ocellar triangle with several short, proclinate setulae (Fig. 2a). Fronto-orbital plate of male with about three irregular rows of fine, medioclinate setae lateral to frontal setae. Seven to 10 frontal setae. Two or 3 upper (= dorsal) reclinate orbital setae, often not clearly differentiated from frontal setae. Proclinate orbital setae absent in male, 2 in female (Fig. 1a–b). Parafacial covered with proclinate, short, fine setae (Fig. 1a–b, 1e–f). Facial ridge straight in profile, with only a few setae above vibrissa on about 1/5–1/4 of its length. Vibrissa arising at about level of lower facial margin. Face concave; lower facial margin not visible in profile. Antenna slightly shorter than facial ridge. Postpedicel 3.0–3.5 times as long as pedicel. Arista bare, thickened on basal 2/5–1/2. First aristomere not longer than wide (usually shorter); second aristomere 1–2 times as long as wide. Genal dilation well developed. Gena in profile very narrow, about 0.10–0.15 times as high as compound eye (height measured in the same vertical plane as height of head). Postocular setae fine, relatively long and slightly bent anteriorly. Occiput flat, with 1–2 rows of black setulae behind postocular row. Prementum not more than 2 times as long as wide; palpus well developed, apically covered with setulae, often strongly clavate in female.

Thorax (Figs 1a–d, 2b–d): Postpronotum with 4 setae; 3 strongest, basal, arranged in a line (Fig. 2b). Scutum with 2–3 posthumeral setae, 1 + 3 supra-alar (first postsutural supra-alar seta at most as long as a notopleural seta), 0–1 + 3 intra-alar, 3 + 4 dorsocentral, 3 + 3 acrostichal setae. General hair-like setulae of scutum fine, relatively long and erect. Prosternum laterally setose. Proepisternal depression bare. Two katepisternal setae (the posterior one larger). Katepimeron setulose along its length. Anepimeral seta at most half as long as posterior katepisternal seta. Scutellum wider than long, covered with long, fine, erect setulae. Three pairs of marginal scutellar setae (basal, subapical, apical) (Figs 1c–d, 2c); basal and subapical setae about equal in size; apical pair shorter, crossed and sub-horizontal. Scutellum without discal setae. Anterior and posterior lappets of metathoracic spiracle unequal in size.

Legs: Preapical anterodorsal seta of fore tibia about as long and stout as preapical dorsal seta. Mid tibia with 1 anterodorsal seta. Hind tibia with a row of moderately spaced, comb-like anterodorsal setae (Fig. 2e); 2 dorsal preapical setae. Preapical posteroventral seta of hind tibia not differentiated. Claws about as long as fifth tarsal segment in male (Fig. 2f), considerably shorter in female.

Wing (Figs 1a–d, 2d): Bend of vein M usually obtuse. Cell r_4+5_ open. Section of M between crossveins r-m and dm-cu longer than section between dm-cu and bend of M. Section of M between dm-cu and bend of M shorter than post-angular section of M. Vein R_4+5_ with a single setula at base dorsally and 0–1 ventrally. Lower calypter large and strongly convex, especially along its lateral and posterior margins (Fig. 2d).

Abdomen (Fig. 1a–d): Syntergite 1+2 with mid-dorsal depression extending to hind margin. Tergites 1+2 and 3 with a pair of fine median marginal setae, sometimes not differentiated from the general abdominal setae, and a pair of lateral setae; tergite 4 with a row of marginal setae; tergite 5 with scattered weak setae. Tergites 3 and 4 without median or lateral discal setae.

**Figure 3.**
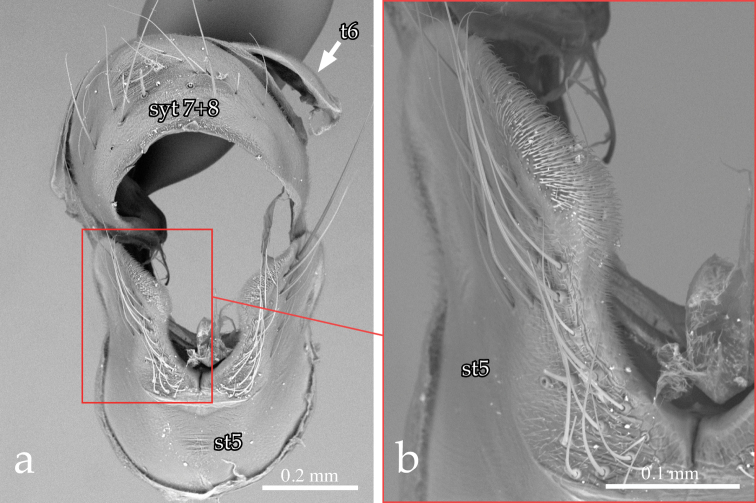
*Neoethilla* gen. n.*ignobilis* (male, New Mexico) [**st5** = sternite 5; **syt7+8** = syntergite 7+8; **t6**  = tergite 6] **a** sternite 5 and syntergite 7+8 **b** detail of left lobe of sternite 5.

**Figure 4.**
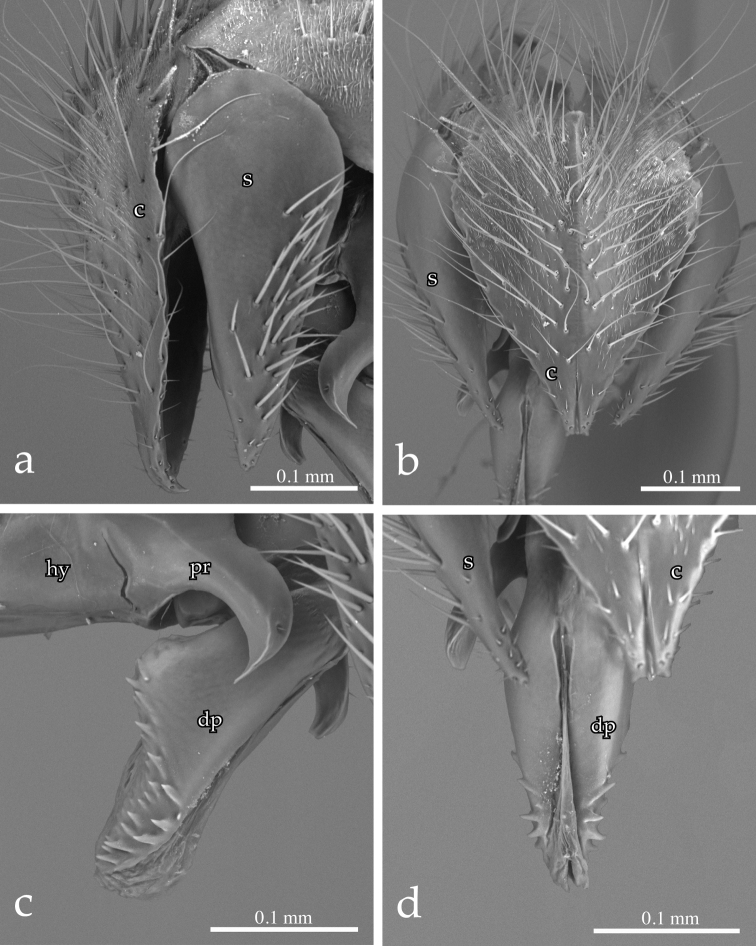
*Neoethilla* gen. n.*ignobilis* (male, New Mexico) [**c** = cerci; **hy** = hypandrium; **dp** = distiphallus; **pr** = pregonite; **s** = surstylus] **a** cerci and right surstylus in lateral view **b** epandrial complex in posterior view **c** distiphallus and pregonite in left lateral view **d** distiphallus in dorsal view.

**Figure 5.**
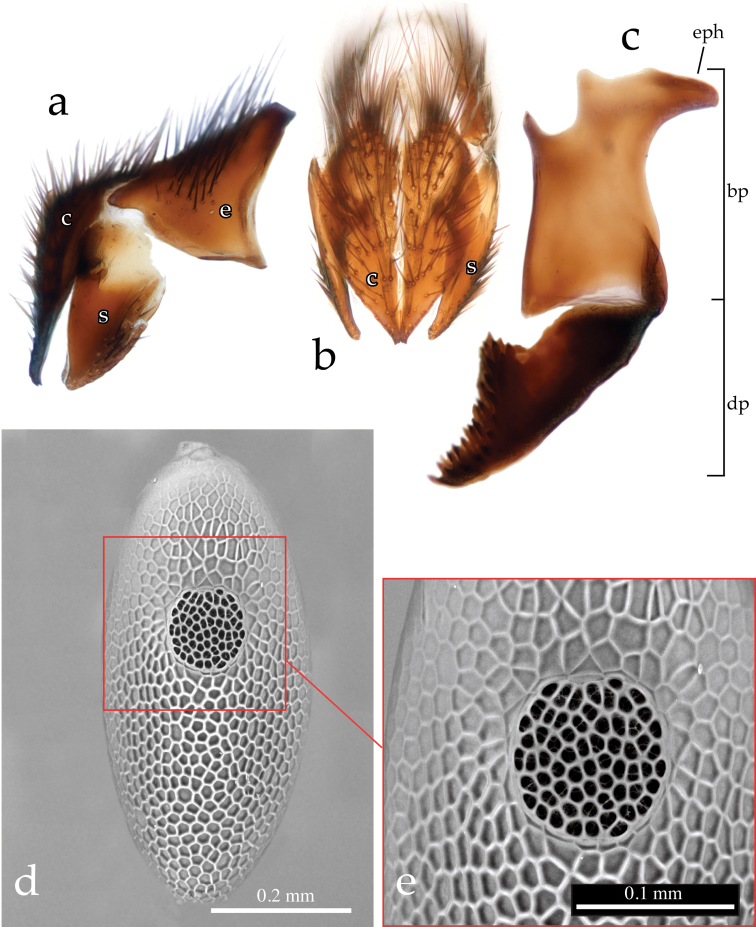
*Neoethilla* gen. n. *ignobilis* **a–c** terminalia (male, Idaho) [**c** = cerci; **bp** = basiphallus; **dp** = distiphallus; **e** = epnadrium; **eph** = epiphallus] **a** epandrial complex in lateral view **b** epandrial complex in posterior view **c** phallus in lateral view **d–e** egg in dorsal view (Massachusetts) **d** habitus **e** detail of *operculum*.

Male terminalia (Figs 3a–b, 4a–d, 5a–c): Sternite 5 with deep median cleft, outer lobe almost truncate along posterior margin (Fig. 3a–b). Tergite 6 large, plate-like (not divided into two sclerites nor indentate on posterior edge), bare; tergite 6 separated from tergite 5 and segment 7+8 by membrane. Cerci (Figs. 4a–b, 5a–b) almost flat, wide in posterior view (sub-ovoid), not fused medially (i.e., longitudinal medial suture complete), distally very slightly divided (Figs 4b, 4d, 5b). Surstylus long, wide and sub-triangular in lateral view, distal tip sometimes slightly bent posteriorly (Figs 4c, 5a). Posterior half of lateral surface of surstylus with several stout setae. Pregonite and postgonite not fused. Pregonite strongly recurved and pointed. Processi longi long, slender and well separated from each other. Epiphallus stout, well sclerotized, attached to basal portion of basiphallus (Fig. 5c). Connection between basiphallus and distiphallus strongly sclerotized (Fig. 5c). Lateroventral sclerites of distiphallus well developed, strongly sclerotized with robust spines lateroventrally (Figs 4a–b, 5c).

Female terminalia. Ovipositor short, not telescopic as in Winthemiini.

Egg. Plano-convex macrotype unembryonated; long-oval in dorsal view; anterodorsally operculate (Fig. 5d–e). Dorsal, convex surface of egg characterized by a strong polygonal micro-sculpturing.

#### Remarks.

*Neoethilla* is superficially similar to *Winthemia* because it has an enlarged compound eye covered with thick and long ommatrichia and parafacial covered with fine setulae. Moreover, *Neoethilla* and *Winthemia* both have a short first postsutural supra-alar seta, a comb-like row of anterodorsal setae on hind tibia and a fully setulose katepimeron. *Neoethilla* is distinguishable from *Winthemia* (i) in having the three strongest basal setae of postpronotum arranged in a line, (ii) in lacking the lateral scutellar setae and (iii) in having processi longi of male terminalia long, slender and well separated from each other. Females of *Neoethilla* have a short ovipositor and a dorsally operculate plano-convex egg. These characters, together with the strongly convex lower calypter, suggest that *Neoethilla* has an ethilline affiliation. Within this tribe the new genus is characterized by the following combination of character states: (i) parafacial fully setulose, (ii) gena very narrow (0.10–0.15 times as high as compound eye), (iii) ocellar setae absent or very reduced, (iv) three strongest basal postpronotal setae arranged in a line, and (v) lateral scutellar setae missing.

##### Included species and examined specimens

### Neoethilla ignobilis

(van der Wulp, 1890) comb. n.

http://species-id.net/wiki/Neoethilla_ignobilis

Exorista ignobilis van der Wulp, 1890: 71. Type material examined: holotype ♂ (BMNH): HOLO- / TYPE [disc with red border] // ♂ // Amula, / Guerrero, / 6000 ft. / Aug. H.H.Smith. // B.C.A. Dipt.II. / Exorista / ignobilis, / v.d.W. // Central America. / Pres. by / F.D.Godman. / O.Salvin. / 1903-172. // HOLOTYPE / of *Exorista* / *ignobilis* Wulp / designated 1998 / D.M. Wood.
Winthemia antennalis Coquillett, 1902: 115, **syn. n.** Type material examined: holotype ♀ (USNM): Los Angeles / Co., CAL. // JULY // Collection / Coquillett // Type / No 6222 / U.S.N.M. [red label] // Winthemia / antennalis / Coq.

#### Other material examined.

UNITED STATES. *Arizona*: 1 ♂, Cochise County, Chiricahua Mountains, 1760 m, 31°52'26.1"N, 109°13'55.8"W, 18.VIII.2007, P. Cerretti leg. (MZUR). 1 ♂, [Cochise County], Chiricahua Mountains, Ash Spring, 1860m, 31°52.3'N, 109°14.7'W, 20–21.IX.2004, J.E. O’Hara leg. (CNC). 3 ♀♀, [Cochise County], Huachuca Mountains, Ramsey Canyon, 1680 m, 2.V.1967, D.M. Wood leg. (CNC). 1 ♂, Cochise County, [Huachuca Mountains], Ida Canyon, 1800 m, 31°23.5'N, 110°19'W, 22–24.X.2001, G. & M. Wood leg. (CNC). 1 ♂, Coconino County, 2 km w. Sunset Crater National Monument, 2100 m, 23–24.VII.1982, J.E. O’Hara leg. (CNC). *California*: 2 ♂♂, [Riverside County], Elsinore, 13.VII.1931 (CNC). *Idaho*: 1 ♂, Craters of the Moon National Monument, 8.VII.1965, D.S. Horning Jr. leg. (CNC). *Maryland*: 1 ♂, Calvert County, Port Republic, 21.VIII.2001, D.M. Wood leg. (CNC). *Massachusetts*: 1 ♀ (with egg), Plymouth County, Onset, 29.VI–6.VII.1983, W.J. Morse leg. (CNC). *New Mexico*: 1 ♂, Grant County, 21 km n. Silver City, Cherry Creek Campground, 2100 m [not 2250 m or 7400’ as printed on some labels from this locality], 32°54.8'N, 108°13.6'W, 16–19.VIII.1982, J.E. & W.M. O’Hara leg. (CNC). 1 ♂, same data except 14–16.VIII.1983, J.E. O’Hara leg. (CNC). 1 ♂, same data except 26.V.1991 (CNC). 2 ♂♂, same data except 16.IX.1994 (CNC). 2 ♂♂, same data except 15–16.VIII.1999 (CNC). 2 ♂♂, Grant County, n. Silver City, Gomez Peak (hilltop), 2200 m, 32°50'N, 108°17'W, 23.IX.2004, J.E. O’Hara leg. (CNC). 1 ♂, same data except 24.IX.2004 (CNC). 1 ♂, same data except 27.VIII.2006 (CNC). 1 ♂, same data except 9.VIII.2007 (CNC). 1 ♂, same data except 4.V.2010 (CNC). 1 ♂, same data except 11.X.2010 (CNC). 8 ♂♂, same data except 9.VIII.2011 (CNC). 6 ♂♂, same locality, 12.VIII.2007, P. Cerretti leg. (MZUR). 5 ♂♂, Grant County, n. Silver City, Eighty Mountain (hilltop), 2275 m, 32°50.9'N, 108°18.0'W, 26.VIII.2006, J.E. O’Hara leg. (CNC). 1 ♂, same data except 15.VIII.2007 (CNC). 3 ♂♂, same locality, 15.VIII.2007, D.M. Wood leg. (CNC). 1 ♀, Grant/Sierra County, 63 km e. Silver City, Emory Pass, 2500 m, 32°54'N, 107°45'W, 12.VIII.2007, G.& M. Wood leg. (CNC). 3 ♂♂, Torrance Co., 14 km s. Cedarvale, North Peak (hilltop), 2250 m, 34°16.4'N, 105°43.5'W, 7.VIII.2007, J.E. O’Hara leg. (CNC). *South Carolina*: 1 ♂, Oconee Co., Sumter [as “Sumpter”] National Forest, Hunt Camp, 1.V.1997, M. Wood leg. (CNC).

#### Distribution.

Neotropical: Mexico (Guerrero); Nearctic: widespread in continental United States (O’Hara and Wood 2004).

## Discussion

The Ethillini are a small tribe of Exoristinae formerly thought to be exclusively Old World in distribution. Most Ethillini share a number of character states with several members of Winthemiini but the relationships between these tribes remain unclear as they have not been investigated with a rigorous cladistic approach. The tribe can be defined by the following character states:

(1) Lower calypter strongly convex (Fig. 2d) – this is a very rare condition in the Tachinidae (as well as in other Calyptratae) and could arguably be considered apomorphic. Excluding *Mycteromyiella* Mesnil, 1966 and *Calliethilla* Shima, 1979, whose systematic placements in Ethillini are questionable (see also Crosskey 1984; Shima 1979; Cerretti 2012), all the other ethilline taxa share this character state. Interestingly, a very convex lower calypter is also present in a few species of *Winthemia*. This could easily be interpreted as convergence if not for the fact that Winthemiini also share other character states with Ethillini (see below),

(2) katepimeron usually entirely covered with fine setulae – within the subfamily Exoristinae this character state is shared with practically all known Winthemiini, a few *Exorista* and the exoristine genus *Crassicornia* Kugler, 1980 (cf. Cerretti et al. 2012; Cerretti 2012). Important exceptions exist for *Atylomyia* Brauer, 1898 and a few *Amnonia* Kugler, 1971 where the katepimeron is often entirely bare, and

(3) female ovipositor short (i.e., non-telescopic) – this could be a plesiomorphic condition compared to the long and telescopic one present in all Winthemiini.

In addition to the above states, all examined female ethillines have a macrotype plano-convex egg of white color. All the Exoristinae, most Phasiinae and the Eutheriini also have a plano-convex egg, or at least traces of a primitive planoconvex shape. This type of egg thus represents a plesiomorphic condition for Ethillini. It is worth noting that Ethillini show two reproductive strategies (Tschorsnig 1988): females with a short ovisac that lay unincubated eggs and females with a long ovisac in which eggs are stored until embryogenesis is complete (*Paratryphera*-group, see below).

Worldwide the following genera fall well within the above limits: *Amnonia*, *Atylomyia*, *Ethilla*, *Ethylloides* Verbeke, 1970, *Gynandromyia* Bezzi, 1923, *Nemorilloides* Brauer & Bergenstamm, 1891, *Neoethilla* gen. n., *Paratryphera* Brauer & Bergenstamm, 1891, *Phorocerosoma* Townsend, 1927, *Prosethilla* Herting, 1984, and *Zelindopsis* Villeneuve, 1943. As mentioned above, *Calliethilla* and *Mycteromyiella* have a questionable ethilline affiliation because of the lack of the distinctive convex lower calypter.

The grasshopper parasitoids *Gynandromyia* and *Phorocerosoma* and the genus *Zelindopsis* (*Phorocerosoma*-group) share the following: postpronotum with five setae, with the three basal strongest arranged in a triangle; female sternites 4–6 very large and exposed from the ventrolateral margins of the corresponding tergites; and egg surface dorsally smooth with two aeropilar areas, irregular in shape. The number and disposition of postpronotal setae are exactly the same as in nearly all the Winthemiini, and the egg characteristics are shared with several other Exoristinae. However, the features of the female abdominal sternites appear to be unique within the Exoristinae and may represent a strong apomorphy in support of the monophyly of this group of ethilline genera. Interestingly, males of *Mycteromyiella* share with males of several species of the *Phoroserosoma*-group the presence of distinctive spots of setulae and microtrichia on the ventral side of abdominal tergites 4 and 5, and also its members are parasitoids of Phasmatodea (Shima 1976), which are currently considered the living sister-group of the Orthoptera (cf. Grimaldi and Engel 2005).

All the remaining ethilline genera (Ethillini s. str.) share the following, probably derived, character states:

(1) postpronotum with the three strongest, basal, setae arranged in a line (Fig. 2b),

(2) male tergite 6 bare, wide and not indented antero-medially,

(3) male segment 7+8 very wide and platiform (cf. Tschorsnig 1985), and

(4) egg distinctly operculate (Tschorsnig 1988) (Fig. 5d–e).

The presence of operculate eggs is a very rare condition in tachinids. In most Exoristini an operculum is delineated by a line at the anterior end of the egg (cf. Wood 1972; Cerretti 2010), in Eutherini an oval “window” is situated on the dorsal surface of the egg immediately in front of its posterior end (Herting 1966), while in Ethillini s. str. an operculum is situated on the dorsal surface of the egg in its anterior half or in the middle (cf. Tschorsnig 1988; Tschorsnig and Richter 1998). Such substantial differences in the shape and position of the operculum in these groups suggest that the three types may have evolved independently and thus be non-homologous. Based on the four states listed here we consider the Ethillini s. str. to be a monophyletic lineage.

Finally, two probably monophyletic groups may be identified within Ethillini s. str. (cf. Tschorsnig 1985; Cerretti 2010). The first is the *Paratryphera*-group, composed of *Amnonia*, *Atylomyia* and *Paratryphera*. These taxa share the following character states:

(1) long ovisac for storing fully embryonated eggs,

(2) pregonite and postgonite at least partly fused basally,

(3) connection between male sternite 6 and segment 7+8 on right side very narrow,

(4) intermedium (cf. Tschorsnig 1985) not differentiated,

(5) epiphallus light-colored and attached to apical portion of basiphallus,

(6) ventrolateral sclerites of distiphallus greatly reduced, and

(7) dorsal connection between basiphallus and distiphallus almost membranous.

The second probably monophyletic group within Ethillini is the *Ethilla*-group, composed of *Ethilla*, *Nemorilloides*, *Prosethilla* and *Neoethilla* gen. n. These taxa share the following character states:

(1) short ovisac, females lay unembryonated eggs,

(2) pregonite and postgonite not fused,

(3) connection between male sternite 6 and segment 7+8 on right side very wide,

(4) intermedium very large,

(5) epiphallus massive and attached to basal portion of basiphallus (Fig. 5c),

(6) ventrolateral sclerites of distiphallus heavily sclerotized and covered with spinulae (Figs 4c–d, 5c), and

(7) dorsal connection between basiphallus and distiphallus heavily sclerotized (Fig. 5c).

We transfer the New World species *Exorista ignobilis* van der Wulp to the Ethillini and assign it to the new genus *Neoethilla* based on its distinctive characteristics. *Neoethilla* may share a sister-group relationship with *Prosethilla*, as evidenced by its lack of lateral marginal setae on the scutellum and similar egg morphology (operculum, microsculpture and shape very similar in these genera, judging from figures of *Prosethilla* by Tschorsnig (1988)). *Neoethilla* is clearly distinguishable from *Prosethilla* by its very large compound eye that occupies most of the head in lateral view (gena very narrow, compared to 1/3 or more of vertical eye height in *Prosethilla*), parafacial entirely covered with fine setulae (as in *Winthemia*), and ocellar setae not (or just slightly) differentiated from other setulae on the ocellar triangle.

## Supplementary Material

XML Treatment for Neoethilla

XML Treatment for Neoethilla ignobilis
